# Does continuous endurance exercise in water elicit a higher release of ANP and BNP and a higher plasma concentration of FFAs in pre-obese and obese men than high intensity intermittent endurance exercise? – Study protocol for a randomized controlled trial

**DOI:** 10.1186/1745-6215-14-328

**Published:** 2013-10-10

**Authors:** Klaus Karner-Rezek, Beat Knechtle, Matthias Fenzl, Joeri Gredig, Thomas Rosemann

**Affiliations:** 1 Private University of the Principality of Liechtenstein, Triesen, Principality of Liechtenstein; 2 Institute of General Practice and for Health Services Research, University of Zurich, Zurich, Switzerland; 3 Gesundheitszentrum St. Gallen, St. Gallen, Switzerland; 4 Swiss Olympic Medical Center, Medizinisches Zentrum, Bad Ragaz, Switzerland

**Keywords:** Obesity, ANP, BNP, EPOC, Exercise intensity, Water immersion

## Abstract

**Background:**

Atrial natriuretic peptides (ANP) and Brain natriuretic peptides (BNP) stimulate fat cell plasma membrane receptors. They are potent lipolytic agents on isolated fat cells from subcutaneous adipose tissue. The physiological effects of continuous endurance exercise on ANP release and plasma free fatty acids (FFA) concentrations have been well described. The enhancement of fat metabolism using high intensity intermittent exercise protocols has been assessed in more recent investigations. The combined effects of endurance exercise and water immersion on ANP and FFA plasma concentration and the magnitude of excess post-exercise oxygen consumption (EPOC) might be further enhanced by choosing the most effective exercise protocol. Exercise modalities may play a significant role in the future prevention and treatment of obesity.

**Methods/design:**

The two testing trials will be performed according to a randomized and cross-over design. Twenty healthy sedentary pre-obese and obese class-1 men will be scrutinized with regard to their metabolic responses to continuous exercise in water and to high intensity endurance exercise in water. Both trials will be matched for energy expenditure. After preliminary testing, the tests will be conducted as repeated measurements. The two different exercise protocols will be compared. The aims of the study are to investigate (1) whether continuous endurance exercise or high intensity intermittent endurance exercise in water elicits both a higher release of ANP and BNP and a higher plasma concentration of glycerol and (2) to determine whether continuous endurance exercise in water or a high intensity intermittent endurance exercise in water would lead to a more pronounced short term (two hours) EPOC effect.

**Discussion:**

If our hypothesis would be confirmed, the most effective exercise protocol based on the combined effects of high intensity endurance exercise and water immersion on ANP and BNP release and glycerol plasma concentrations can be identified. Moreover, the magnitude of the EPOC effect can be augmented. Our study would provide a major contribution for creating optimized exercise modalities in the prevention and treatment of obesity.

**Trial registration:**

Current controlled trials, ISRCTN95488515

## Background

Lipolysis of adipose subcutaneous tissue is controlled by a diversity of inhibiting and stimulatory pathways. Catecholamines, natriuretic peptides (NPs) and insulin are considered as the main endocrine related factors in the regulation of lipolysis [1]. In fat cells lipolysis is activated through stimulation of β-adrenergic receptors (ARs) and inhibited through activation of the α_2_-adrenergic receptor [2]. The simultaneous activation of both receptors modulates the intracellular cyclic adenosine monophoshate (cAMP)-dependent protein kinase, leading to the phosphorylation and activation of perilipin and hormone-sensitive lipase [2,3].

Compared to lean subjects, obese subjects seem to have impaired exercise-induced subcutaneous adipose tissue lipolysis [3,4] - in particular obese subjects with insulin resistance or type-2 diabetes. This is mainly attributed to the activation of antilipolytic α_2_-adrenergic receptors during exercise. These α_2_-adrenergic receptors largely outnumber β-adrenergic receptors in subcutaneous adipocytes and α_2_-adrenergic receptors density correlates with fat cell size [2,3]. In obese subjects complete blockade of fat cell receptors, however, does not suppress the exercise-induced lipolysis entirely [1-4]. Other lipolytic pathways thus stimulate lipolysis and contribute to the regulation of lipid mobilization in humans [5].

A growing body of evidence suggests that NPs play a role in the regulation of lipid metabolism via the control of adipose tissue lipolysis [6]. Atrial natriuretic peptides (ANPs) stimulate fat-cell plasma membrane receptors bearing intrinsic guanylylcyclase activity and increase intracellular levels of cyclic adenosine monophosphate (cGMP), which activates a cGMP-dependent protein kinase (PKG). ANPs are potent lipolytic agents on isolated fat cells from subcutaneous adipose tissue [3]. Thus, the ANP-dependent lipolytic pathway appears to be involved in the enhancement of lipolysis in subcutaneous adipose tissue and is responsible for the residual mobilization under adrenergic receptor blockade [3]. The fall in plasma insulin during exercise could also contribute to the enhancement of lipolysis due to the reduction of its antilipolytic effect.

The ability of mammals to resist weight gain and body fat accumulation is very closely tied, both genetically and pharmacologically, to the capacity to expand the number and activity of brown adipocytes within white fat depots, and, in most cases, this is also correlated with improved blood glucose levels, insulin sensitivity, and body composition [7]. The conversion of white to brown-like adipocytes operates under physiological conditions in humans; NPs could be included in the list of factors contributing to the physiological control of energy dissipation. [8].

In addition, the very recent demonstration that NPs increase oxidative capacity and energy uncoupling in human skeletal muscle opens interesting perspectives. [8]. Activation of NP signaling in human skeletal muscle enhances mitochondrial oxidative metabolism and fat oxidation. NP could contribute to exercise training-induced improvement in skeletal muscle fat-oxidative capacity in humans [9]. Ideally, NP signaling in skeletal muscle, enhanced by exercise, may trigger favorable metabolic adaptations to increase fat oxidation, alleviate lipotoxicity, and enhance insulin sensitivity [8]. Chronic upregulation of adipose tissue NP receptor type C (NPR-C) expression may increase NP clearance, reduce circulating NP levels, and contribute to obesity-related cardiovascular and metabolic disorders. The potential relevance of insulin-induced upregulation of NPR-C on circulating NP levels in humans is still unclear and needs to be investigated in further long-term observations [8].

Endurance training improves ANP as well as beta-adrenergic-receptor-mediated lipid mobilization in the subcutaneous adipose tissue of obese subjects [1-4]. The physiological effects of continuous endurance exercise on ANP release and plasma free fatty acid (FFA) concentrations have been well described [1,3]. The further enhancement of fat metabolism using high-intensity intermittent exercise protocols has been the focus of more recent investigations [10-13]. High intensity intermittent exercise provides more potent metabolic stimuli, leading to increased lactate and catecholamine levels. The accompanying rise in plasma glycerol concentrations suggests that fat stores may supply a significant amount of energy during this form of exercise [13]. Investigations have shown that circulating ANP concentrations increased 2- to 3-fold during short exercise bouts of increasing intensities [10]. ANP may act as a relevant physiological agent controlling exercise-induced lipolysis in endurance exercise consisting of repeated bouts [3,14]. The magnitude of the excess post-exercise oxygen consumption (EPOC) effect is also dependent on the type of the preceding exercise protocol. A curvilinear relationship between the magnitude and the intensity of the exercise bout has been reported [15].

Water immersion attenuates adrenaline and noradrenaline and augments ANP release during exercise [16]. Even though water immersion blunts exercise-induced sympatho-adrenal activation, lipid mobilization and lipid oxidation rates are maintained or even improved [16]. Free fatty acid concentrations are increased; glucose and lactate measurements are decreased [16]. The combined effects of endurance exercise and water immersion on ANP and FFA plasma concentration and the magnitude of EPOC might be further enhanced by choosing the most effective exercise protocol. Exercise modalities may play a significant role in the future prevention and treatment of obesity.

The first aim of this study is to investigate whether continuous endurance exercise or high-intensity intermittent endurance exercise in water elicits both a higher release of ANP and a higher plasma concentration of FFAs. The second aim is to determine if continuous endurance exercise in water or high-intensity intermittent endurance exercise in water leads to a more pronounced short term (that is, 2 hours) EPOC effect.

## Methods

### Trial design

**Figure 1:**
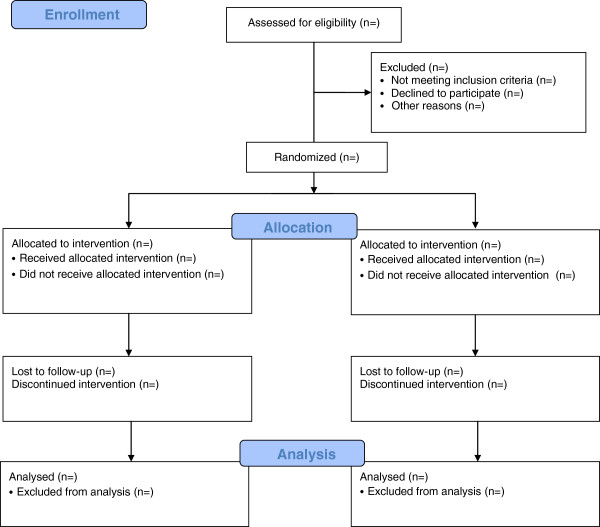
Trial design.

The two testing trials will be performed according to a randomized crossover design. Subjects will be allocated to one of the two intervention groups according to a prepared randomization list and will at this time be unaware of group to which they are allocated. Implementation of the random allocation sequence will be done via central telephone to provide allocation concealment. The flow chart of the study is described in Figure 1.

### Participants

Eligible participants will be recruited via advertisements on the websites and magazines such as *FIT FOR LIFE* and *Schweizerische Adipositas-Stiftung*. A sample of 20 healthy sedentary pre-obese and obese class-1 men with a body mass index (BMI) ranging from 30.0 kg/m^2^ to 34.9 kg/m^2^ according to the World Health Organization (WHO) 2004, will be scrutinized with regard to their metabolic responses to continuous exercise in water and to high-intensity endurance exercise in water. Participants will be informed about potential risks and the trial process both verbally and in written form. Ethical approval and consent was obtained by the Ethics Committee in St. Gallen, Switzerland.

### Inclusion criteria for participants

The inclusion criteria are: male gender (differences in estrogen concentrations between men and woman result in greater reliance on fat oxidation during exercise in women; between the different phases of the female menstrual cycle substrate utilization also varies); BMI between >30.0 kg/m^2^ and <34.9 kg/m^2^; age >18 years and <50 years; verbal and written information of the participants, signed declaration of consent; and no cardiovascular risk (completion of the Physical Activity Readiness Questionnaire (PAR-Q)).

### Exclusion criteria for participants

The exclusion criteria are: female gender; cardiac insufficiency >New York Heart Association (NYHA) stage 1; continuous arrhythmia; respiratory obstruction; high blood pressure ( >140/90 mmHg); BMI <30.0 kg/m^2^ and >34.9 kg/m^2^; age <18 years and >50 years; any medication; or signed declaration of consent missing.

### Intervention

The aims of the study are to investigate (1) whether continuous endurance exercise or high-intensity intermittent endurance exercise in water elicits both a higher release of ANP and BNP and a higher plasma concentration of glycerol, and (2) to determine if continuous endurance exercise in water or high-intensity intermittent endurance exercise in water leads to a more pronounced short term (that is, 2 hours) EPOC effect.

### Overview

Twenty healthy sedentary pre-obese and obese class-1 men with BMI ranging from >30 kg/m^2^ to <34.9 kg/m^2^ according to WHO 2004, will be scrutinized with regard to their metabolic responses to continuous exercise in water and to high-intensity endurance exercise in water. Both trials will be matched for energy expenditure. After preliminary testing, the tests will be conducted as repeated measurements, with 72 hours of separation time in between. The two different exercise protocols will be compared.

### Environmental conditions and preparation of the subjects

All trials will be conducted under standardized environmental conditions in regard to the temperature of air and water (that is, the thermoneutral zone on land between 23°C and 26°C at a relative humidity of 60 to 70%, equivalent to a water temperature of 28°C). Participants will be tested at 8 am after an overnight fast following a standardized two-day diet and will be asked to refrain from any physical exercise on the day before testing. Prescription drugs will be recorded.

### Preliminary testing

Maximum voluntary ventilation (MVV) and forced expiratory volume (FEV_1_) will be assessed initially. The ventilatory threshold 2 (VT2) as defined by Wassermann *et al*. [17] will be determined in a separate testing session at least one week before the experiment. Participants will perform a ramp test on a cycle ergometer until exhaustion. An electrocardiogram (ECG) will be performed and blood pressure will be monitored simultaneously as an ergospirometry. The identification of the VT2 is required in order to assess the defined workloads for the trials. Lean body mass will be determined using dual-energy x-ray absorptiometry (DXA).

### Outcomes

#### Primary outcomes

In addition to ANP and BNP, the primary outcomes are the respiratory exchange rate (RER) and the rating of perceived exertion (RPE). The RER specifies the ratio of carbon dioxide (CO_2_) eliminated to oxygen (O_2_) consumed. The RER depends on the metabolic substrate used for generating energy. Using stoichiometric equations, the RER can be applied to determine the amounts of carbohydrates and fatty acids metabolized. Energy production can also be reliably estimated in the same manner. The RPE method requires that a person subjectively rates how difficult the work is, using a numerical scale that is related to exercise intensity.

#### Secondary outcomes

Secondary outcomes are lactate, catecholamine (that is, adrenaline, noradrenaline, and dopamine), growth hormone, insulin and glycerol, all of which indicate changes in the metabolic process.

### Assessment of effectiveness and proof of evidence

#### Measurement of effectiveness

Effectiveness will be measured by the following methods:

•Gas-exchange open system, breath-by-breath-method (Viasys Jäger Master Screen CPX, PanGas AG, Dagmarsellen, Switzerland)

•ECG-monitoring by means of suction electrodes (Viasys Jäger Master Screen CPX, PanGas AG, Dagmarsellen, Switzerland)

•Continuous heart-rate monitoring (Polar-S-810 heart rate monitor)

•measuring of the blood pressure according to Riva-Rocci via inflatable cuff on the upper arm

•Rating of perceived exertion (Borg-scale)

•Sampling of blood (ANP, BNP, glycerol, FFAs, lactate, catecholamine (adrenaline, noradrenaline, dopamine), growth hormone, insulin, and glucose).

#### Proof of evidence

Proof of evidence will be provided by the following parameters:

•RER, VO_2_ and CO_2_

•ANP concentration (radioimmunoassy)

•Glycerol concentration (radiometric determination)

•Free fatty acids (FFAs) (enzymatic determination)

•Lactate (photometric determination)

### Assessment of safety

#### Safety parameters: measuring methods and times

Exclusion criteria for the ergospirometry are subjective disorders (stenocardia, dyspnea, dizziness, muscular exhaustion), variations in the ECG during the ergospirometry (ST-depression > 0.2 mV, ST-elevation > 0.2 mV, dysrhythmia), pathological changes in blood pressure (no rise or fall in blood pressure during the examination, blood pressure values >250/130 mm Hg) and a measured heart rate (HR) above the age-based maximum (HRmax = 220 – age in years).

The oxygen pulse as a correlate of the stroke volume is a relevant clinical safety parameter. When workload increases, a reduced stroke volume leads to stagnation of the oxygen pulse followed by a slow drop of the values measured. Therefore, the oxygen pulse is fundamental when estimating the myocardial function. The oxygen absorption will also be evaluated in regard to changes in the workload. Cardiovascular disease leads to low measured values, and performance limitations become observable. The respiratory reserve allows for estimation of pulmonary ventilation. Healthy subjects are not limited in their performance by pulmonary factors. The respiratory reserve is still sufficient (approximately 50% MVV when exercising at their maximum. Pulmonary diseases lead to approximation of the respiratory reserve to the MVV, indicating a pulmonary limit. Respiratory gases, ECG and blood pressure will be assessed, allowing for differentiated identification of cardiac reactions. In the event of a severe and life-threatening incident, the ethics committee and the sponsor will be informed.

Blood samples will be taken by laboratory assistants who are familiar with the operating procedures and safety standards. During the trial blood samples will be taken every 15 minutes (continuous endurance exercise) or after each exercise step (high-intensity intermittent endurance exercise). Moreover, one blood sample will be taken pre- and post-exercise. Viollier laboratories (in-house) will process and analyze the blood samples.

### Data collection

Data collection is described in detail in the Methods section.

### Statistical methods

Figures will be presented as means ± standard error of the mean (SEM). Differences in outcomes among the groups will be tested by analysis of covariance (ANCOVA). For statistical significance (*P* <0.05), multiple comparisons will be adjusted for using the Tukey test.

Our assumptions for the power calculation are mainly based on the results of Fenzl *et al*. [18] who recently reported an increase of ANP from 29.8 ± 3.4 pmol/l to 82.5 ± 8.3 pmol/l. Assuming an increase from 35 to 55 with a SD of 10, we would need eight patients in each group to achieve 90% power at the 1% significance level (two-sided test). With an assumed drop-out rate of two in each group, we need 20 persons in total.

Hormone values that are expected to rise during the recovery period of the exercise include catecholamines, ANP, BNP, glycerol and free fatty acids. Differences in these hormones between high-intensity intermittent exercise protocols compared to less intense protocols have not yet been analyzed; studies investigating intermittent bouts of exercise results were in contrast to moderate continuous exercise of the same duration. Different scores relative to the contribution of fat oxidation for repeated trials (77.6% ± 2.7%) versus single trials (62.1% ± 5.7%) were computed in the study of Goto *et al*. [11]. A test would require less than 10 subjects in each group, assuming a similar response to water immersion. Matsuo *et al*. [19] demonstrate that EPOC was greater when high-intensity interval training was conducted compared to aerobic types of exercise. For the power calculation, eight subjects in each group should be included into the study.

### Trial-specific preventive measures and duties

Participants will be informed about any risks and the trial process both verbally and in written form.

### Duties on the part of the investigator

It is hereby confirmed that ethical and scientific criteria as well as quality standards in terms of planning, trial procedure, monitoring, analysis and documentation of the trial will be fully observed and carried out in accordance with the protocol. All rights of the participants will be respected and the results of the trial will be handled correctly. The investigator and applicant are responsible for the wellbeing and health protection of the participants. The investigator also commits to carry out the trial according to the trial protocol and to report and document deviations to the sponsor and the ethics committee.

### Statement regarding damage coverage

The sponsor compensates the participant for any damage suffered during the clinical trial. In order to cover possible costs, insurance will be procured. The sponsor of the trial approves full compensation to all participants of the trial also beyond the coverage of the liability insurance.

### Ethical principles

#### Evaluation of the risk-benefit ratio

The paper will be considered a preventive contribution for obese persons. The evidence of the aqua-intervention with regard to weight reduction and weight stabilization will be worked out. Existing interventions will be further improved according to the criteria of evidence-based medicine by choosing the right exercise protocol. Health-related recommendations can only be made once the physiological basis for substrate utilization has been established and a qualitative verification of the fat oxidation has been carried out. Measurements of the partial pressure of oxygen and carbon dioxide in water are routine, and so is the measurement of the arterial oxygen saturation. The examination rooms in the Medical Center permit standardized relaxation and defined exercise tests on the Reha Aquabike. All the planned interventions are justifiable and reasonable from a medical point of view. The measurements are non-invasive and painless. In water every participant will be protected from possible drowning by an air-filled ruff. So far, no accident has been reported worldwide.

#### Patient informed consent

Previous to study participation patients receive written and spoken information about the content and extent of the planned study, for instance, about potential benefits for their health and potential risks. In the case of acceptance, they sign the informed consent form.

#### Description of risks

Exercise testing is not without risk. Under controlled conditions an increase of heart rate and oxygen uptake is concomitant with the increased workload. In the event of undetected myocardial disease the heart may not be sufficiently supplied with blood. Symptoms such as sudden pallor, sweating, chest discomfort, or nausea have to be reported immediately.

The risk of undetected coronary heart disease and sudden death will be reduced to a large extent by the preliminary medical check-up. The risk increases with age, therefore, the age limit is 50 years. A defibrillator will be available. Moreover, the standard emergency equipment required for ergometry laboratories will be available. Trials will only be conducted with healthy subjects without detected risk factors. A doctor on-call will be available in the medical center at all times, who will be informed about the upcoming exercise test. Taking blood samples is part of the discomfort and risk involved in ergospirometry. In addition to a little twitch and subsequent small hematomas, circulatory instability may occur. Blood samples will be taken while the subject is in a semi-reclined position, which stabilizes the circulation. Infection, thrombosis or lesions of tissue or nerves are rare side effects and can be almost excluded when blood samples are taken by well-trained staff. An inflatable ruff prevents drowning if the subject becomes unconscious during the trial. The subjects will be continuously monitored.

#### Benefit for the subject

Subjects will not be given compensation in money. The individual results of the ergospirometry, ECG, and pulmonary function test will be discussed in detail with the subjects; extensive consultations about adequate health-oriented training recommendations will be given. Subjects will receive meaningful additional information about their current level of performance by parameters assessed by invasive methods (lactate, et cetera). Thus subjects will receive reliable information concerning their current state of health. Moreover, recommendations on the individual effects of water immersion and exercise modalities will allow participants a more targeted use of water as a medium. Until now, recommendations have been given only on an empirical basis, following across-the-board dosage criteria.

#### Vote of the ethics committee

The study protocol was approved by the ethics board of the Kanton St. Gallen on March 14, 2011 (EKSG 11/026/2B).

### Quality control and quality assurance: description of measures

#### Data security/disclosure of original documents

Gathered data will be stored in anonymized form only and will be used exclusively for this trial. No conclusions can be drawn about the person affected. Each subject will be assigned an identification code, which will be kept on a separate datasheet. This code will be used during the entire trial. Personal data allowing identification of the subjects will be deleted. In addition to all members of the research staff who are bound to their professional obligation to discretion, all members of the ethics committee may have access to the data at any time. All documents and data storage media containing encrypted personal data will be stowed in locked storage. The laptop computer used in the trial will be protected by a password in order to prevent access from unauthorized persons. A personal firewall will be installed by the internal information technology (IT) provider. Data will be stored in databases, mainly as Excel files. The trial can be reconstructed using the encrypted data. Original files, such as protocols, will be stored with care. All relevant data will be transferred to the digital data-entry form. Thus, all results of the trial can be retraced for a five-year period on the internal server, which is protected by a password. All data on the laptop computer will be erased by the internal IT provider.

## Trials status

The recruitment of subjects has not yet started.

## Abbreviations

ANP: Atrial natriuretic peptide; AR: Adrenergic receptor; BMI: Body mass index; BNP: Brain natriuretic peptide; cAMP: Cyclic adenosine monophosphate; cGMP: Cyclic adenosine monophosphate; CO2: Carbon dioxide; DXA: Dual-energy x-ray absorptiometry; ECG: Electrocardiogram; EPOC: Post-exercise oxygen consumption; FEV1: Forced expiratory volume; FFA: Free fatty acids; HR: Heart rate; IAT: Individual anaerobic threshold; MVV: Maximum voluntary ventilation; NP: Natriuretic peptide; NNPR-C: Natriuretic peptide receptor type C; NYHA: New York Heart Association; O2: Oxygen; PAR-Q: Physical Activity Readiness Questionnaire; PKG: CGMP-dependent protein kinase; RER: Respiratory exchange rate; RPE: Rating of perceived exertion; WHO: World Health Organization.

## Competing interests

The authors declare that they have no competing interests.

## Authors’ contributions

KK, BK, MF and JG designed the basic idea, KK, BK and MF drafted the manuscript, JG and TR planned the data analysis. All authors read and approved the final manuscript.
